# Impact of Fecal Microbiota Transplantation on Obesity and Metabolic Syndrome—A Systematic Review

**DOI:** 10.3390/nu11102291

**Published:** 2019-09-25

**Authors:** Zhengxiao Zhang, Valentin Mocanu, Chenxi Cai, Jerry Dang, Linda Slater, Edward C. Deehan, Jens Walter, Karen L. Madsen

**Affiliations:** 1 Division of Gastroenterology, Department of Medicine, University of Alberta, Edmonton, AB T6G 2E1, Canada; zh16@ualberta.ca; 2 Division of General Surgery, University of Alberta, Edmonton, AB T6G 2E1, Canada; vmocanu@ualberta.ca (V.M.); dang2@ualberta.ca (J.D.); 3 Program for Pregnancy and Postpartum Health, Women and Children’s Health Research Institute, University of Alberta, Edmonton, AB T6G 2E1, Canada; ccai1@ualberta.ca; 4 John W. Scott Health Sciences Library, University of Alberta, Edmonton, ON T6G 2E1, Canada; linda.slater@ualberta.ca; 5 Department of Agricultural, Food and Nutritional Science, University of Alberta, Edmonton, AB T6G 2E1, Canadajwalter1@ualberta.ca (J.W.); 6 Department of Biological Sciences, University of Alberta, Edmonton, AB T6G 2E1, Canada

**Keywords:** fecal microbiota transplantation, gut microbiome, obesity, metabolic syndrome, insulin sensitivity, microbial ecology

## Abstract

Fecal microbiota transplantation (FMT) is a gut microbial-modulation strategy that has been investigated for the treatment of a variety of human diseases, including obesity-associated metabolic disorders. This study appraises current literature and provides an overview of the effectiveness and limitations of FMT as a potential therapeutic strategy for obesity and metabolic syndrome (MS). Five electronic databases and two gray literature sources were searched up to 10 December 2018. All interventional and observational studies that contained information on the relevant population (adult patients with obesity and MS), intervention (receiving allogeneic FMT) and outcomes (metabolic parameters) were eligible. From 1096 unique citations, three randomized placebo-controlled studies (76 patients with obesity and MS, body mass index = 34.8 ± 4.1 kg/m^2^, fasting plasma glucose = 5.8 ± 0.7 mmol/L) were included for review. Studies reported mixed results with regards to improvement in metabolic parameters. Two studies reported improved peripheral insulin sensitivity (rate of glucose disappearance, RD) at 6 weeks in patients receiving donor FMT versus patients receiving the placebo control. In addition, one study observed lower HbA1c levels in FMT patients at 6 weeks. No differences in fasting plasma glucose, hepatic insulin sensitivity, body mass index (BMI), or cholesterol markers were observed between two groups across all included studies. While promising, the influence of FMT on long-term clinical endpoints needs to be further explored. Future studies are also required to better understand the mechanisms through which changes in gut microbial ecology and engraftment of microbiota affect metabolic outcomes for patients with obesity and MS. In addition, further research is needed to better define the optimal fecal microbial preparation, dosing, and method of delivery.

## 1. Introduction

Obesity and metabolic syndrome (MS) are among the greatest health epidemics of the 21st century. In 2016 alone, nearly 2 billion adults were overweight and over 650 million adults were obese [1]. As a group of obesity-related metabolic abnormalities, the prevalence of MS has reached about 25% of the world’s adult population [2,3]. The economic impact of this epidemic is overwhelming, with the annual global cost of obesity estimated at $2.0 trillion in 2012, nearly surpassing the economic costs of smoking, war, and terrorism [4]. With rates of obesity rapidly climbing, the management of obesity and its metabolic complications is at the forefront of modern research.

Therapeutic strategies aimed at managing obesity and MS include lifestyle interventions, pharmacologic therapies, and bariatric surgery [5,6,7,8,9,10,11]. Effective, lifestyle modifications are resource-intensive and are prone to weight recidivism for the majority of patients [5,6,7]. Pharmacologic therapies, on the other hand, are costly and associated with significant side effects with long-term use [8,9,10]. Bariatric surgery is currently the most effective sustained treatment for obesity yet is associated with significant operative risks and complications [12,13]. Novel, safe, and effective therapeutic approaches are, therefore, required to address the growing obesity epidemic.

The gut microbiome is an ecosystem of an estimated 10~100 trillion microorganisms residing in the human gastrointestinal tract [14,15,16]. Alterations in gut microbial composition and function, commonly referred to as a “dysbiosis” have been associated with a variety of human diseases including *Clostridioides difficile* infection (CDI), irritable bowel disease, inflammatory bowel disease, type 2 diabetes, cardiovascular disease, and most recently obesity and MS [17,18,19]. Dysbiosis of the gut microbiota has been defined as a shift in gut bacterial communities, relative to healthy individuals, towards an unbalanced microbial composition often with more “inflammatory” microbes (i.e. Proteobacteria), reduced diversity and decreased levels of beneficial metabolites such as short-chain fatty acids [20,21,22]. Although growing evidence from animal disease models have described potential causative relationships between an altered gut microbiota and obesity, whether the gut dysbiosis in human obesity is causative or occurs as a consequence still needs to be elucidated [19,23,24]. For example, gut microbiota analysis of genetically obese mice, compared with their lean counterparts has revealed a lower gut microbial genes diversity and higher ratio of Bacteroidetes to Firmicutes. Germ-free mice colonized with this obesity-associated microbiota have been found to have increased body fat and energy harvest compared to mice colonized with lean donor microbiota [25,26]. A more recent study of 154 human twins revealed that obesity was associated with reduced gut bacterial diversity, Bacteroidetes abundance, levels of butyrate and propionate, and elevated branched-chain amino acids. Transfer of fecal content from human twins of different obesity phenotypes to germ-free mice resulted in the mice adopting their human donor phenotypes [27].

Fecal microbiota transplantation (FMT) is a microbial-based strategy that aims to restore the disrupted gut microbial ecosystem [28,29,30]. Over the past few years, FMT has been widely investigated in CDI, a gut dysbiosis-associated disease [31,32,33]. Recent findings have demonstrated that FMT is a highly effective and robust therapy for recurrent CDI and reversing microbial dysbiosis such as an increase in gut bacterial diversity, and decrease in Proteobacteria relative abundance [34,35]. Given the evidence of potential causation between gut microbiota and obesity in animal studies, attempts have been made to transplant gut microbiota from lean and healthy donors into obese and MS recipients in human trials. However, to date, the clinical benefits of using FMT to rebuild gut microbial ecosystems in patients with obesity and MS are not well established.

The goal of the present systematic review was to appraise the current literature and provide an overview of the effectiveness and limitations of FMT as a potential therapeutic strategy for obesity and MS.

## 2. Materials and Methods

A systematic search and retrieval of records was performed in accordance with the Preferred Reporting Items for Systematic Reviews and Meta-Analyses (PRISMA) guidelines [36]. The review was registered with the International Prospective Register of Systematic Reviews (PROSPERO; Registration no. CRD42019129646). A number of deviations from the analysis proposed in the registered PROSPERO protocol were made due to the significant limitations of included data. We intended to conduct a meta-analysis for the key outcomes and use the Grading of Recommendations Assessment, Development and Evaluation (GRADE) tool to assess the certainty across studies for each health outcomes. However, because included studies only reported outcomes on a small numbers of patients with a limited clinical utility, we reported only descriptive summaries of selected outcomes.

### 2.1. Eligibility Criteria

The PICOS (population, intervention, comparison, outcome and study design) framework was used to guide this systematic review. The population of interest was adult subjects with obesity and/or MS. The intervention was fecal microbiota transplantation (FMT), defined as the administration of a solution of fecal matter from a donor into the intestinal tract of a recipient in order to directly change the recipient’s microbiota to confer a health benefit [37,38]. Patients receiving allogenic FMT (the fecal samples come from the human donors) through different modalities (i.e., colonoscopy, enteric tube, or enemas) were all permitted, as were studies that used either single or pooled donor FMT. Our key outcomes of interest included changes in body mass index (BMI), dysglycemia, lipid metabolism, hypertension, waist circumference, gut microbial composition and their associated metabolites. All interventional and observational studies were eligible. Case reports, abstracts, letters, narrative or systematic reviews were excluded.

### 2.2. Information Sources

A structured search of MEDLINE, EMBASE, Cochrane Library, CINAHL, Web of Science Core Collection, Scopus, and ProQuest Dissertations and Theses Global was performed by a research librarian (LS) on 10 December 2018. Reference lists of included papers and relevant reviews were screened for additional relevant papers. Language restrictions were not applied. See online supplementary documents for complete search strategies.

### 2.3. Study Selection and Data Extraction

Titles and abstracts of relevant articles were first assessed by two independent reviewers (ZZ and VM). Studies meeting initial screening criteria by at least one reviewer were selected for full text review. Two independent reviewers examined all full text articles for eligibility (ZZ and VM). Studies published in languages other than English, Chinese, or French that were considered to be potentially important were translated using Google Translate for full-text screening. Data was extracted independently by two reviewers (ZZ and VM) and discrepancies were resolved by consensus or through assessment by a third independent reviewer (KM). Relevant data from all publications were extracted independently into an Excel document and cross-examined for accuracy. Study characteristics were evaluated for type of study design, year of study, and country of origin. Population variables included number of patients, age, sex, hip and waist circumference, BMI, blood pressure, insulin sensitivity parameters, fasting plasma glucose (FPG) levels, hemoglobin A1c levels (HbA1c), total cholesterol, high-density lipoprotein cholesterol (HDL-C), low-density lipoprotein cholesterol (LDL-C), and triglycerides (TG). FMT-specific variables were also extracted including gut microbial changes, donor stool processing, and delivery methods.

### 2.4. Data Synthesis

The findings are summarized narratively as included studies had significant limitations making any pooled estimate of the effect size of limited clinical value.

### 2.5. Assessment of Risk of Bias

Two reviewers (ZZ and CC) independently assessed the risk of bias of the individual studies following the Cochrane Handbook [39] for RCT studies. All studies were assessed for potential sources of selection bias, performance bias, reporting bias, detection bias, attrition bias and ‘other’ sources of bias. The risk of bias was assessed as low, high or unclear.

## 3. Results

### 3.1. Search Results

**Figure 1:**
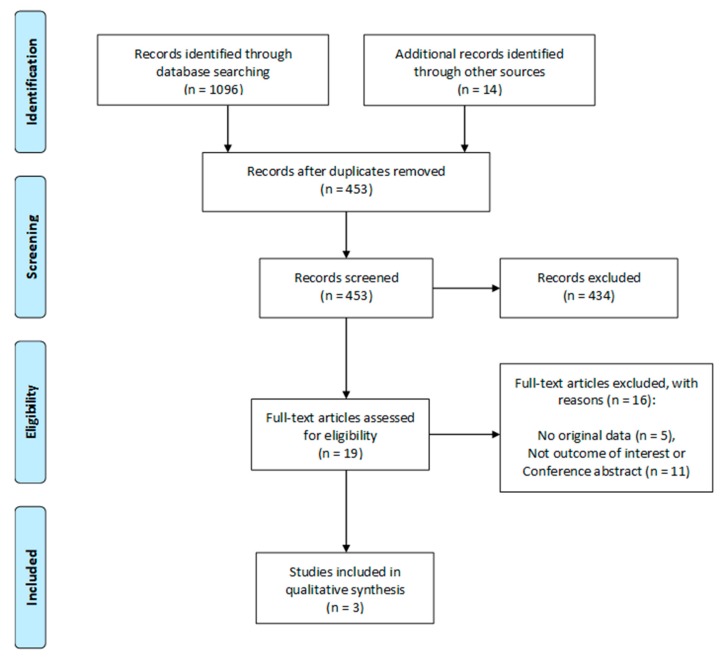
Preferred Reporting Items for Systematic Reviews and Meta-Analyses (PRISMA) flow diagram.

A preliminary database search of available literature revealed 1096 potential articles. After screening titles and abstracts, and removing duplicates, 23 studies were selected for full-text review (Figure 1). Review of the full text articles identified three randomized placebo-controlled trials (RCTs) [40,41,42] that were eligible for inclusion in the final systematic review. No other eligible observational studies were found.

### 3.2. Study Characteristics and Baseline Demographics

A total of three RCT studies with 76 patients were included (Table 1). Of the total population at baseline, the weighted mean age was 53 ± 9 years, the weighted mean BMI was 34.8 ± 4.1 kg/m^2^, and only male participants were involved. Follow-up ranged from 2 to 18 weeks. The weighted means of FPG was 5.8 ± 0.7 mmol/L, HbA1c was 39.2 ± 5.0 mmol/mol, LDL-C was 3.5 ± 1.2 mmol/L, HDL-C was 1.1 ± 0.3 mmol/L, TG was 1.4 ± 0.8 mmol/L, and total cholesterol was 5.3 ± 1.3 mmol/L (Table 2). Of the participants, 45 (59%) received donor FMT while 31 (41%) received a placebo consisting of autologous stool [40,41,42].

### 3.3. Fecal Microbiota Transplantation (FMT) Donor and Delivery Method

FMT stool donors and processing varied across all studies (Table 3). All three RCTs used a naso-duodenal delivery method for FMT following a bowel lavage with polyethylene glycol. Vrieze, et al. [40] and Kootte, et al. [41] used single unpooled stool samples from different lean donors, whereas Smits, et al. [42] used single unpooled stool samples from different vegan donors.

### 3.4. Assessment of Risk of Bias

Results of the assessment of risk of bias in included studies are summarized in online supplemental Figures S1 and S2. Unclear reporting about random sequence generation and allocation concealment were the main reasons for unclear risk of bias, while selective reporting was the main reason for high risk of bias. Other potential sources of bias were rarely suspected. The overall risk of bias assessment of included studies was low.

### 3.5. FMT and Metabolic Outcomes

Studies reported mixed results with regards to improvement in dysglycemia metabolic parameters. Vrieze, et al. [40] and Kootte, et al. [41] reported that peripheral insulin sensitivity (rate of glucose disappearance, RD) increased at 6 weeks in patients receiving donor FMT versus patients receiving the placebo control. Hepatic insulin sensitivity (endogenous glucose production, EGP) was further assessed in two studies but no statically differences were found. Kootte, et al. [41] observed a lower HbA1c level in patients who received donor FMT at 6 weeks than in patients receiving the placebo control. However, this study indicated the patients who received donor FMT did not show difference in HbA1c or insulin sensitivity (RD) after 18 weeks [41]. This finding suggests that the observed short-term benefit of FMT on dysglycemia was not maintained long-term. In contrast, three of included studies indicated no significant difference in the FPG levels between patients receiving donor FMT and control patients.

Included studies demonstrated no differences between patients receiving donor FMT and patients receiving placebo with regards to cholesterol profile, including the levels of total cholesterol, HDL-C, LDL-C and TG. Vrieze, et al. [40] and Kootte, et al. [41] also reported no significant differences on BMI between patients receiving donor FMT and patients receiving placebo followed at 6 weeks.

### 3.6. FMT Influences Microbiome Composition and Derived Metabolites

Two studies indicated no difference in α-diversity, assessed by Shannon index, of the gut microbiome between patients receiving donor FMT or patients receiving the placebo [41,42] (Table 4). FMT significantly increased the relative abundance of 16 microbial species including the butyrate-producing species *Roseburia intestinalis* [40,43] and *Clostridium* spp. Comparison between FMT and placebo groups also demonstrated significant differences in several other microbial species, including the oxalate-converting species *Oxalobacter formigenes*, and the donor-enriched *Clostridium* spp. [40,41,42]. Based on the observed insulin sensitivity response (RD), Kootte et al. split the FMT-treated subjects into two groups: responders and non-responders. Within the responders, a significant increase in the relative abundance of *Akkermansia muciniphila* compared to baseline was observed [41].

Fecal short chain acids (SCFAs) shifts in recipients following donor FMT were inconsistent. After the donor FMT, one study reported a decrease in the fecal acetate and butyrate levels in obese patients [40], whereas another study reported an increase [41]. With respect to other microbial derived metabolites, FMT did not induce any shift in the plasma or urine trimethylamine N-oxide (TMAO) levels, or fecal secondary bile acids (i.e., Deoxycholic acid and Lithocholic acid) in the obese recipients [41,42].

## 4. Discussion

### 4.1. Effect of FMT on the Metabolic Parameters

To the best of our knowledge, the present systematic review is the first to evaluate FMT as a potential therapeutic strategy for obesity and MS. FMT is associated with improvements in RD and HbA1c at 6 weeks. The beneficial effects of FMT on RD and HbA1c were not maintained long-term in the single study evaluating 18-week outcomes [41]. While promising, the clinical impact of these observed short-term benefits in insulin sensitivity are not clear and require further evaluation. In contrast to markers of dysglycemia, other important clinical parameters of obesity and MS including BMI, FPG, TG, HDL-C and LDL-C showed no improvement between groups. Taken together, FMT in patients with obesity and MS showed a short-term benefit on insulin sensitivity but did not confer a benefit with regards to other clinical parameters.

Transplantation of the entire fecal microbiota from the healthy donor to the recipient could influence host metabolism by modulating microbial composition and/or functions [28,29,30]. In terms of short-term improvement in insulin sensitivity, subjects receiving FMT demonstrated increases in relative abundance of *Ruminococcus bromii* and *Roseburia intestinalis* [40], species that are well-known as dietary fiber degraders and butyrate producers, respectively [43,44], which may play a role in improving insulin sensitivity through regulation of glucagon-like peptide-1 [45,46,47] and intestinal gluconeogenesis [48]. Patients receiving FMT also demonstrated increased species belonging to *Clostridium* compared to the placebo group [40,42]. A recent murine study revealed that a reduction in Clostridia and increases in *Desulfovibrio* abundance were associated with disorders of lipid absorption and insulin resistance. This may be due to defective T follicular helper cell responses and inappropriate IgA targeting of Clostridia [49]. Additionally, one study indicated an increase in the mucin-degrading species *Akkermansia muciniphila* in those subjects that had increased insulin sensitivity following FMT [41]. Human and animal studies have demonstrated that *A. muciniphila* is closely associated with improvements in insulin sensitivity [50,51,52,53], and the beneficial effects may be due to a microbial-induced increased intestinal level of endocannabinoids and epithelial toll-like receptor2, which regulates gut barrier function and inflammation [50,54]. However, these shifts in bacterial species following transplantation were not consistent across all studies.

SCFAs, which are the primary end products of microbial dietary fiber fermentation, have been shown to regulate glucose metabolism, innate immunity, and energy homeostasis [55,56]. However, there was no consistent change following transplantation in fecal acetate and butyrate across the studies.

**Figure 2:**
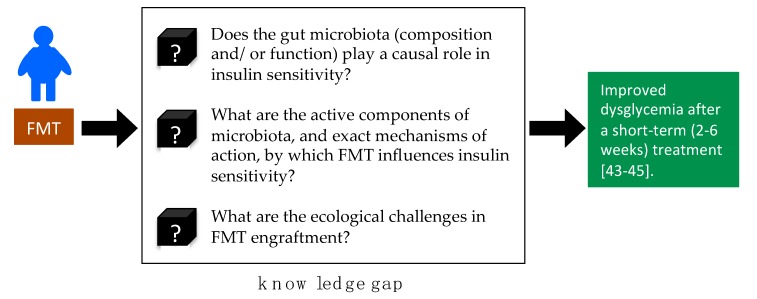
Mechanistic aspects of fecal microbiota transplantation (FMT) in obesity and metabolic syndrome (MS) requiring targeted research.

FMT is one strategy to manipulate the entire gut microbiota based on the idea of the microbiome (composition and/or function) as a causal agent in disease. However, causal relationships between the human gut microbiome and obesity-associated disease are not clear [57]. According to our review, while the shift in specific microbes may contribute to insulin sensitivity improvement, the characterizations vary and the nature of the microbiome being manipulated by FMT appears to be unstable. Therefore, the active component of FMT and exact mechanisms by which FMT influences MS remains unknown (Figure 2).

Recent human metagenomic microbiome studies revealed that the individuals with obesity have less protein gene family richness and abnormal functions encoded genes related to enterohemorrhagic *Escherichia coli* pathogenicity, lipopolysaccharide biosynthesis and acetate production [58]. Applying advanced methodology such as multi-omics techniques to identify more causal relationships between the microbiome and metabolic pathways will aid in further understanding the mechanisms by which FMT affects the gut microbiome in obesity and MS.

### 4.2. The Ecological Challenges of the FMT on Obesity and its Related Metabolic Disorders

One remaining question is why FMT showed limited improvement in the clinical parameters of obesity and MS [41]. In our review, the microbial α-diversity (Shannon index) of obese patients was not improved by FMT (Table 4) [41,42]. It is not clear, however, whether successful microbiota engraftation is key to a successful clinical response following FMT. Human gut bacterial communities can be self-regulating and resilient to change [59]. Although the studies found differences in the gut bacterial composition (i.e. lower relative abundance of *Clostridium* cluster XIVa and higher Bacteroidetes in the recipients) [40], the α-diversity was not distinct between the MS recipients and lean donors at baseline [42]. Microbial communities with higher diversity are considered more resilient and better able to exploit resources efficiently, thereby lowering the level of available resources and providing less opportunity for bacterial invasions [60]. While possibly beneficial in healthy states, this resilience may serve as a barrier to the reversal of gut dysbiosis by FMT in patients with MS. Therefore, FMT microbiota might have a much lower degree of engraftment in patients with obesity and MS than in patients with CDI, whose gut microbiome diversity is profoundly disturbed and thus provides minimal colonization resistance [38,61]. Overall, the gut ecosystem resilience to invasion by new species [62] may pose a challenge to successful FMT engraftment and minimize potential benefits to metabolic parameters.

Secondly, gut dysbiosis could be influenced by inflammation, diet, and other host environmental exposures [63]. Obesity is a complex condition that is associated with adipose-mediated, systematic low-grade chronic immune dysfunction [64]. The host immune system has been shown to influence the colonization niche of the gut microbiota through a variety of mechanisms, including the production of antimicrobial peptides including IgA [63,65,66]. Diet has also been well reported to impact gut microbiota composition and functional activity [43,67,68], largely through increasing available nutrients to bacterial niches [69]. Therefore, diet and chronic inflammation may act as host environmental pressures that select for microbes possessing the necessary adaptive traits to colonize the gut [38], thus propagating gut dysbiosis [70]. Without addressing the entire host milieu, a similar dysbiotic microbiome would likely be re-selected even after microbial transplantation. One potential solution to improve engraftment, sustain reversal of dysbiosis, and further improve metabolic outcomes would be to implement microbiome-targeted dietary strategies following FMT (i.e., prebiotic supplementation).

### 4.3. FMT in Clinical Practice

One challenge in analyzing data related to the use of FMT as a therapeutic modality for obesity and MS was the variation in the completeness of information in the studies on stool preparation, delivery and dosing. Three of the included RCTs did not clearly describe whether anaerobic or aerobic stool preparation was used. Anaerobic preparation in FMT may be necessary to increase the bacterial viability and engraftment success of strict anaerobes [71,72]. For instance, *Faecalibacterium prausnitzii* and *Akkermansia muciniphila*, the two species highly associated with metabolic health, are both oxygen sensitive [73,74]. FMTs with anaerobically prepared stools were used in a recent RCT study that showed high remission effectiveness in treating ulcerative colitis (UC) (odds ratio, 5.0 [95% confidence interval (CI), 1.2–20.1]) [75]. However, the benefit, if any, of anaerobic preparation of donor stool for FMT in obese and MS has not been studied yet.

Secondly, a systematic review of FMT for CDI treatment has indicated that different routes of delivery including duodenal (upper intestine), colonoscopy, and enema (lower intestine), gave rise to different remission rates [76]. To date, only one delivery protocol has been tested for MS [40,41,42] and uncertainty remains regarding the optimal FMT delivery route. Conversely, a systematic review of FMT for treatment of ulcerative colitis (UC) reported similar remission rates between upper and lower intestinal delivery [72]. Since only one delivery protocol has been tested for MS, uncertainty remains about the effect of the delivery route on the efficacy of FMT.

In addition, further work is needed to understand the dose and duration of therapy needed to maximize the therapeutic effect of FMT while optimizing patient tolerance and compliance [77]. In the case of UC, one ongoing study (Clinicaltrials.gov NCT03006809) aims to compare the various stool doses and routes of FMT administration. However, the dosage response of FMT (grams of donor stool and/or microbial load in the stool) for improving metabolic disorders is likewise unknown.

### 4.4. Limitations and Strengths

Our study has several significant limitations. All studies were performed solely in male subjects, despite female obesity rates worldwide being higher than males as of 2016 [1]. Sex is recognized implicitly as an important factor in a variety of common disorders including autoimmune, metabolic, cardiovascular and psychiatric diseases [78,79,80]. This may affect study validity and generalizability. The differences in sex may also influence the immune–microbe interactions [81,82,83,84]. Therefore, future FMT studies should include female subjects in trials and assess for potential sex-mediated differences in clinical outcomes.

The study population in our systematic review was primarily composed of individuals with class I obesity making our findings potentially less generalizable to individuals with more severe weight or metabolic abnormalities. However, long-term studies assessing patients with more extreme metabolic abnormalities should be conducted only once a clinical benefit is demonstrated given the increased clinical risk in delaying bariatric surgery.

Our systematic review findings were also limited by the small number of studies and the small trial sample sizes. Furthermore, the studies were conducted primarily by one research group and may not reflect expected outcomes across global populations. Importantly, only one study [44] published the raw microbiome data, making it impossible to perform meta-analyses across the individual microbiome datasets. We advocate for FMT studies to publish gut microbiome sequencing data to allow for future high-quality aggregate bioinformatic analysis.

Despite these limitations, we report the most comprehensive analysis of the role of FMT on metabolic outcomes for patients with obesity and MS. We utilized a comprehensive search strategy to identify and summarize a multitude of metabolic parameters, microbial data, and sources of bias for all included studies. Taken together, we identify that FMT may be associated with short-term statistically significant improvements in dysglycemia (insulin sensitivity) for patients with obesity and MS. The degree to which these improvements are clinically relevant, however, has yet to be determined.

### 4.5. Future Direction

While promising, the influence of FMT on long-term clinical endpoints needs to be explored. Further studies are also required to better understand the mechanisms through which changes in composition and function of gut microbiome affect metabolic outcomes for patients with obesity and MS. Given that the human gut microbiota is a complex ecosystem, the ecological challenges of engraftment such as microbiota resilience, competitive exclusion, and host environmental filtering should be considered when developing future FMT studies. Dietary intervention would be one option for maintaining the FMT engraftment and efficacy in regulating the metabolic response. Lastly, more research is needed to better define the optimal fecal microbial preparation, dosing, and method of delivery.

## Figures and Tables

**Table 1:** Baseline demographics of included studies.

Study	Country	Follow up (Weeks)	Study Arms	Patients (n)	Age (Years)	Sex (%Female)	Obesity Criteria	Metabolic Syndrome Criteria
Vrieze et al. 2012 [40]	NL	6	FMT	9	47 ± 12	0	BMI > 30	Waist circumference >102 cm and a FPG level >5.6 mmol/L
			Placebo	9	53 ± 9	0		
Koote et al. 2017 [41]	NL	6 and 18	FMT	26	54 (49–60)	0	BMI ≥ 30	National Cholesterol Education Program (NCEP)
			Placebo	12	54 (49–58)	0		
Smits et al. 2018 [42]	NL	2	FMT	10	52 ± 7.4	0	BMI ≥ 30	FPG ≥ 5.6 mmol/L; TG ≥ 1.7 mmol/L; HDL-C < 1.0 mmol/L; blood pressure ≥ 130/85 mm Hg; waist circumference ≥ 102 cm
			Placebo	10	58 ± 8.5	0		

RCT: randomized controlled trials; NL: Netherlands; FMT: fecal microbial transplant; BMI: body mass index; FPG: fasting plasma glucose; HDL-C: high-density lipoprotein cholesterol; TG: triglycerides.

**Table 2:** Metabolic parameters of included studies.

Study			Vrieze et al. 2012 [40]		Koote et al. 2017 [41]		Smits et al. 2018 [42]	
Follow-up (weeks)			6		6		2	
Study Arms			FMT	Placebo	FMT	Placebo	FMT	Placebo
Patients (*n*)			9	9	13	12	10	10
**BMI**	**kg/m^2^**	**BL**	35.7 ± 4.5	35.6 ± 4.5	33.8 (32.5–35.7)	35.8 (33.1–40.4)	33.9 ± 3.9	33.8 ± 4
		**EP**	35.6 ± 4.2	35.7 ± 4.8	33.6 (32.5–35.8)	36.1 (32.5–41.5)	-	-
**SBP**	**mmHg**	**BL**	138 ± 9	140 ± 6	141 (132–154	148 (134–62)	148 ± 12	152 ± 13
		**EP**	132 ± 18	142 ± 24	-	-	-	-
**DBP**	**mmHg**	**BL**	85 ± 6	84 ± 6	90 (78–97)	94 (83–105)	93 ± 10	93 ± 8
		**EP**	83 ± 15	86 ± 18	-	-	-	-
**FPG**	**mmol/L**	**BL**	5.7 ± 0.6	5.7 ± 0.6	5.5 (5.3–6.1)	5.9 (5.5–6.4)	5.8 ± 0.5	6.2 ± 0.9
		**EP**	5.7 ± 0.6	5.7 ± 0.6	5.6 (5.4–6.9)	5.9 (5.7–6.7)	5.9 ± 0.6	6.0 ± 0.6
**HbA1c**	**mmol/mol**	**BL**	39 ± 3.3	40 ± 4.5	40 (36–41)	43 (36–46)	36.9 ± 5.1	38.7 ± 3.6
		**EP**	38 ± 3.6	39 ± 9.0	38 (34–41)	42 (35–46)	37.4 ± 3.5	38.3 ± 3.9
**Insulin**	**pmol/L**	**BL**	74 (40–230)	135 (26–220)	121 (93–143)	107 (80–159)	146 ± 63.7	107 ± 45.5
		**EP**	77 (18–250)	140 (30–287)	103 (81–126)	126 (97–171)	140 ± 67.8	121 ± 76.5
**EGP**	**μmol/kg/min**	**BL**	3.8 (2.9–9.8)	4.6 (2.6–12.1)	4 (3.3–5.1)	4.6 (3.6–5.5)	-	-
		**EP**	3.8 (1.2–7.8)	4.8 (3.9–12.5)	3.8 (3.2–4.5)	4.7 (2.9–5.5)	-	-
**Rd**	**μmol/kg/min**	**BL**	26.2 (12.6–55.1)	18.9 (10.8–35.9)	25.8 (19.3–34.7)	22.5 (19.6–30.2)	-	-
		**EP**	45.3 (10.6–62.0)	19.5 (13.5–33.2)	28.8 (21.4–36.9)	20.8 (17.6–29.5)	-	-
**Total cholesterol**	**mmol/L**	**BL**	4.5 ± 1.2	4.8 ± 0.9	5.5 (4.8–6.6)	5.5 (4.8–6.6)	5.3 ± 0.9	5.3 ± 0.9
		**EP**	4.6 ± 1.2	4.8 ± 0.6	5.4 (4.8–6.3)	5.4 (5.1–5.7)	5.3 ± 0.9	5.0 ± 0.7
**HDL-C**	**mmol/L**	**BL**	1.0 ± 0.3	1.0 ± 0.3	1.1 (0.9–1.4)	1.0 (0.9–1.1)	1.1 ± 0.2	1.2 ± 0.2
		**EP**	1.0 ± 0.3	0.9 ± 0.3	1.1 (1–1.3)	1.0 (0.9–1.2)	1.2 ± 0.2	1.2 ± 0.2
**LDL-C**	**mmol/L**	**BL**	3.1 ± 1.2	2.9 ± 0.6	3.9 (3.2–4.5)	3.7 (3.0–4.8)	3.3 ± 0.7	3.1 ± 1.3
		**EP**	3.0 ± 0.9	2.9 ± 0.6	3.8 (3.1–4.5)	3.5 (3.2–4.1)	3.5 ± 0.8	3.2 ± 0.5
**TG**	**mmol/L**	**BL**	1.4 ± 0.9	1.6 ± 0.9	1.2 (0.9–1.7)	1.3 (1.1–1.8)	1.3 (1–1.6)	1.3 (1.1–1.6)
		**EP**	1.5 ± 1.2	1.8 ± 1.2	1.3 (0.9–1.6)	1.7 (1.2–2.0)	1.3 (1–2.2)	1.0 (0.7–1.5)

FMT: fecal microbial transplant; BMI: body mass index; SBP: systolic blood pressure; DBP: diastolic blood pressure; HbA1c: glycated hemoglobin; FPG: fasting plasma glucose; HDL-C: high-density lipoprotein cholesterol; LDL-C: low-density lipoprotein cholesterol; TG: triglycerides; EGP: endogenous glucose production; Rd: rate of glucose disappearance; BL: baseline; EP: end point. Data are depicted as mean ± SD or median (interquartile range), depending on their original publication.

**Table 3:** Summary of donor stool processing and delivery methods in randomized controlled trials of FMT for obese and metabolic syndrome.

Study	Vrieze et al. 2012 [40]	Koote et al. 2017 [41]	Smits et al. 2018 [42]
**FMT Route**	Nasoduodenal	Nasoduodenal	Nasoduodenal
**Donor stool**	Single unpooled FMT from different lean donors	Single unpooled FMT from different lean omnivorous donors	Single unpooled FMT from different vegan donors
**Stool preparation**	Fresh sample was immediately covered with sterile saline (500 mL, 0.9% NaCl), and stirred in blender (10 min) and filtered twice through metal sieve.	Fresh sample was immediately covered with sterile saline (500 mL, 0.9% NaCl), and stirred in blender (10 min) and filtered twice through metal	Fresh sample was immediately covered with sterile saline (500 mL, 0.9% NaCl), and stirred in blender (10 min) and filtered twice through metal sieve.
**Stool Dose**	Not reported	Not reported	Not reported
**Time to FMT from stool donation**	<6 h	<6 h	<6 h
**FMT replicates**	1	2	1
**FMT infusion time**	30 min	Not reported	30 min
**Adverse event**	N/A	No serious events	No serious events

N/A: not applicable, which indicated that the study did not report whether there were adverse events during the follow-up period.

**Table 4:** Effects of FMT on the gut microbiome composition and associated metabolites.

Study (Sequencing Method)	Fecal Microbiota Changes in Metabolic Syndrome Patients Relative to Donors	Fecal Microbiota Changes within Group after FMT Infusion	Fecal Microbiota Changes in the FMT Group Relative to the Placebo Group	Microbiota Associated Metabolites Changes Post-FMT Infusion
**Vrieze et al. 2012 [40]** (HITChip microarray)	↑ *Bacteroidetes* ↓ *Clostridium* cluster XIVa	↑ α-diversity (Observed Species) ↑ *Dorea formicigenerans*, ↑ *Clostridium sphenoides*, ↑ *Clostridium symbiosum*, ↑ *Clostridium ramosum*, ↑ *Clostridium nexile*, ↑ *Coprobacillus catenaformis*, ↑ *Ruminococcus gnavus*, ↑ *Ruminococcus lactaris*, ↑ *Ruminococcus callidus*, ↑ *Ruminococcus bromii*, ↑ *Roseburia intestinalis*, ↑ *Aneurinibacillus,* ↑ *Anaerotruncus colihominis*, ↑ *Eubacterium siraeum*, ↑ *Sporobacter termitidis*, ↑ *Oxalobacter formigenes*	↑ *Dorea formicigenerans*, ↑ *Clostridium sphenoides*, ↑ *Clostridium nexile*, ↑ *Coprobacillus catenaformis*, ↑ *Ruminococcus lactaris*, ↑ *Oxalobacter formigenes*	Fecal SCFAs ↓ Acetate ↔ Propionate ↓ Butyrate
**Kootte et al. 2017 [41]** (HITChip microarray)	Not reported	↔ α-diversity (Shannon index). Composition change in Responders ^a^ compared to non-responders ↑ *Eubacterium ventriosum* ↑ *Akkermansia muciniphila* ↑ *Clostridium sporogenes* ↓ *Roseburia intestinalis* ↓ *Bacteroides plebeius*	*Eubacterium siraeum* *Lactobacillus ruminis*	Fecal SCFAs ↑ Acetate ↔ Propionate ↔ Butyrate Fecal Bile Acids ↑ Cholic acid ↔ Chenodeoxycholic acid ↔ Deoxycholic acid ↔ Lithocholic acid
**Smits et al. 2018 [42]** (HITChip microarray)	↔ α-diversity (Shannon index) The distinction in fecal microbiota composition between patients and donors is driven by *Anaerostipes caccae*, *Lachnobacterium* and *Clostridium spp.*	↔ α-diversity (Shannon index)	*Bryantella formatexigens* *Megamonas hypermegale* *Lachnobacterium bovis* *Clostridium cluster XIVa*	TMAO Metabolites ↔ Plasma TMAO ↔ Urinary TMA Excretion ↔ Urinary TMA Excretion

a: based on the observed insulin sensitivity response (rate of glucose disappearance improvement), Kootte et al. split the FMT-treated subjects into two groups: responders and non-responders [41]. HITChip: Human Intestinal Tract chip; Short chain acids: SCFAs; Trimethylamine N-oxide: TMAO; ↔: no significant change (*p* > 0.05); ↑ a significant increase; ↓: a significant decrease.

## Supplementary Materials

The following are available online at https://www.mdpi.com/2072-6643/11/10/2291/s1: Figure S1. Risk of bias of individual study, Figure S2. Risk of bias summary of included studies.
