# Supplementary material for: Impact of Fecal Microbiota Transplantation on Obesity and Metabolic Syndrome—A Systematic Review
Source: Nutrients. 2019 Sep 25;11(10):2291. doi: 10.3390/nu11102291 (PMC6835402; doi:10.3390/nu11102291)
Supplement: Supplementary file 1 [file nutrients-11-02291-s001.pdf]

Online Supplementary Documents

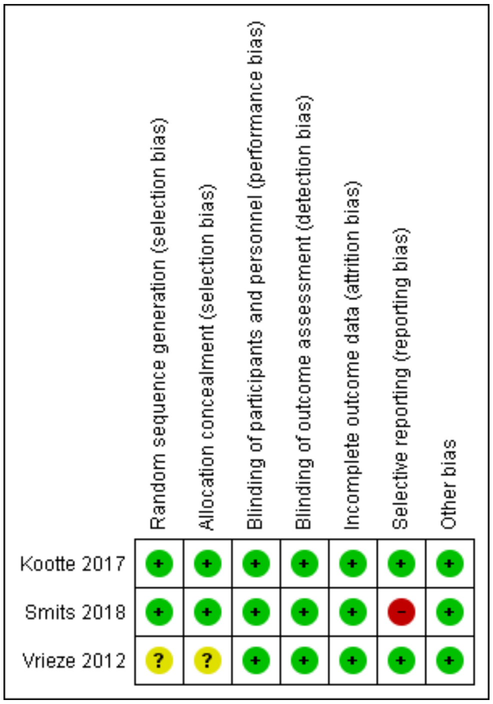

Figure S1. Risk of bias of individual study

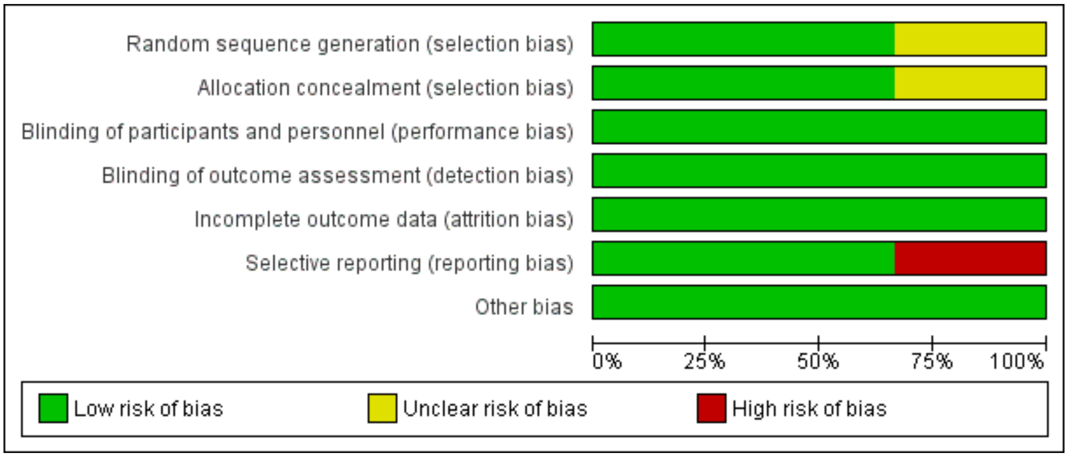

Figure S2. Risk of bias summary of included studies

Search strategies:
The following databases were searched on Dec 10, 2018.

- Ovid MEDLINE(R) and Epub Ahead of Print, In-Process & Other Non-Indexed Citations and Daily 1946 to Current
- Ovid EMBASE, 1974-Current
- Cochrane Library, inception to Current
- CINAHL Plus with Full-text, 1937-current
- Scopus, inception to Current
- Web of Science Core Collection, Inception to Current
- ProQuest Dissertations & Theses Global, Inception to Current

1096 records retrieved in total from all databases; 453 remained after duplicates were removed. No date or language limits were applied.

MEDLINE

1 Fecal Microbiota Transplantation/ or ((f?eces or f?ecal or microbiota or stool or microbiome* or microflor*) adj3 (infus* or transplant* or transfer* or enema* or donor*)).mp.
2 Metabolic syndrome/ or exp Obesity/ or ((metabolic or dysmetabolic or insulin resistan* or reaven) adj2 (syndrome* or disease* or disorder*)).mp. or (obes* or overweight or cardiometabolic risk*).mp.
3 1 and 2
4 animals/ not (humans/ and animals/) or (mouse or mice or murine or rat or rats).mp
5 3 not 4

EMBASE

1 Fecal Microbiota Transplantation/ or ((f?eces or f?ecal or microbiota or stool or microbiome* or microflor*) adj3 (infus* or transplant* or transfer* or enema* or donor*)).mp.
2 Metabolic syndrome x/ or exp Obesity/ or ((metabolic or dysmetabolic or insulin resistan* or reaven) adj2 (syndrome* or disease* or disorder*)).mp. or (obes* or overweight or cardiometabolic risk*).mp.
3 1 and 2
4 animal/ not (human/ and animal/) or (mouse or mice or murine or rat or rats).mp
5 3 not 4

Cochrane Library (Advanced Search/Search Manager)

#1 [mh "Fecal Microbiota Transplantation"] or ((feces or faeces or fecal or faecal or microbiota or stool or microbiome or microflora) near/2 (infus* or transplant* or transfer* or enema* or donor*)):ti,ab,kw
#2 [mh "Metabolic syndrome"] or [mh overweight] or (overweight or obes* or "cardiometabolic risk*" or ((metabolic or dysmetabolic or "insulin resistance" or "insulin resistant" or reaven) near/2 (syndrome* or disease* or disorder*)):ti,ab,kw
#3 #1 AND #2

CINAHL

((feces or faeces or fecal or faecal or microbiota or stool or microbiome or microflora) n2 (infus* or transplant* or transfer* or enema* or donor*))
AND
(MH "Metabolic Syndrome X+") or (MH "Obesity+") or (metabolic or dysmetabolic or "insulin resistan*" or reaven) w2 (syndrome* or disease* or disorder*) or overweight or obes* or "cardiometabolic risk*"
NOT
mouse or mice or murine or rat or rats

Scopus

((feces or faeces or fecal or faecal or microbiota or stool) w/2 (infus* or transplant* or transfer*))
AND
((metabolic or dysmetabolic or "insulin resistan*" or reaven) pre/2 (syndrome* or disease* or disorder*)) or obes* or overweight or "cardiometabolic risk*"
AND NOT
mouse or mice or murine or rat or rats

WOS Core

((feces or faeces or fecal or faecal or microbiota or stool) near/2 (infus* or transplant* or transfer*))
AND
((metabolic or dysmetabolic or "insulin resistan*" or reaven) near/2 (syndrome* or disease* or disorder*)) or overweight or obes* or "cardiometabolic risk*"
NOT
mouse or mice or murine or rat or rats

ProQuest Dissertations & Theses

((feces or faeces or fecal or faecal or microbiota or stool) near/2 (infus* or transplant* or transfer*))
AND
((metabolic or dysmetabolic or "insulin resistan*" or reaven) pre/2 (syndrome* or disease* or disorder*)) or obes* or overweight or "cardiometabolic risk*"
NOT
mouse or mice or murine or rat or rats

References excluded with reasons:

Non-original data

1. Greenhill, C. Microbiota: FMT transiently improves insulin sensitivity. *Nature Reviews Endocrinology* **2017**, *13*, 688, doi:http://dx.doi.org/10.1038/nrendo.2017.137.
2. Rossen, N.G.; MacDonald, J.K.; de Vries, E.M.; D'Haens, G.R.; de Vos, W.M.; Zoetendal, E.G.; Ponsioen, C.Y. Fecal microbiota transplantation as novel therapy in gastroenterology: A systematic review. *World J Gastroenterol* **2015**, *21*, 5359-5371, doi:https://dx.doi.org/10.3748/wjg.v21.i17.5359.
3. Udayappan, S.D.; Hartstra, A.V.; Dallinga-Thie, G.M.; Nieuwdorp, M. Intestinal microbiota and faecal transplantation as treatment modality for insulin resistance and type 2 diabetes mellitus. *Clin Exp Immunol* **2014**, *177*, 24-29, doi:https://dx.doi.org/10.1111/cei.12293.
4. Vrieze, A.; De Groot, P.F.; Kootte, R.S.; Knaapen, M.; Van Nood, E.; Nieuwdorp, M. Fecal transplant: A safe and sustainable clinical therapy for restoring intestinal microbial balance in human disease? *Best Practice and Research: Clinical Gastroenterology* **2013**, *27*, 127-137, doi:http://dx.doi.org/10.1016/j.bpg.2013.03.003.
5. Walters, W.A.; Xu, Z.; Knight, R. Meta-analyses of human gut microbes associated with obesity and IBD. *FEBS Lett.* **2014**, *588*, 4223-4233, doi:10.1016/j.febslet.2014.09.039.

No outcome of interest or conference abstract

1. De Groot, P.F.; Kahn, M.T.; Backhed, F.; Nieuwdorp, M. Faecal microbiota transfer from donors post bariatric surgery does not improve insulin sensitivity in metabolic syndrome subjects. *Diabetologia* **2016**, *Conference: 52nd Annual Meeting of the European Association for the Study of Diabetes, EASD 2016. Germany. Conference Start: 20160912. Conference End: 20160916. 59*, S172-S173, doi:10.1007/s00125-016-4046-9.
2. Fischer, M.; Kao, D.; Kassam, Z.; Smith, J.; Louie, T.; Sipe, B.; Torbeck, M.; Xu, H.; Ouyang, F.; Mozaffarian, D., et al. Stool Donor Body Mass Index Does Not Affect Recipient Weight After a Single Fecal Microbiota Transplantation for Clostridium difficile Infection. *Clinical Gastroenterology and Hepatology* **2018**, *16*, 1351-1353, doi:http://dx.doi.org/10.1016/j.cgh.2017.12.007.
3. Fischer, M.; Sipe, B.; Torbeck, M.; Xu, H.P.; Kassam, Z.; Allegretti, J.R. DOES FECAL MICROBIOTA TRANSPLANTATION FROM AN OBESE DONOR LEAD TO WEIGHT GAIN? A CASE SERIES OF 70 RECIPIENTS. *Gastroenterology* **2017**, *152*, S1004-S1004.
4. Fischer, M.; Torbeck, M.; Cook, G.; Mazur, S.; Phelps, E.; Sipe, B.; Rex, D.K.; Vuppalanchi, R. Weight change after fecal microbiota transplantation (FMT) is not associated with donor body mass index (BMI). *Am J Gastroenterol* **2015**, *1)*, S585, doi:http://dx.doi.org/10.1038/ajg.2015.294.
5. Nieuwdorp, M. Fecal transplantation as a metabolic treatment in humans. *Obesity Facts* **2016**, *1)*, 14, doi:http://dx.doi.org/10.1159/000446744.
6. Saffouri, G.; Pardi, D.; Kashyap, P.; Khanna, S. Body mass index changes after fecal microbiota transplant for recurrent clostridium difficile infection. *Am J Gastroenterol* **2016**, *111 (Supplement 1)*, S103, doi:http://dx.doi.org/10.1038/ajg.2016.352.
7. Rodriguez Jimenez, D.M. Impact of Basal Diet on Obesity Phenotype of Recipient Mice Following Fecal Microbiome Transfer from Obese or Lean Human Donors. M.S., Utah State University, Ann Arbor, 2018.
8. Smith, J.D.; Roach, B.; Hassanzadeh Keshteli, A.; Kao, D.H. Donor Body Mass Index (BMI) does not impact recipient BMI following fecal microbiota transplantation for recurrent clostridium difficile infection. *Journal of the canadian association of gastroenterology* **2018**, *1*, 476-478, doi:10.1093/JCAG/GWY008.276.

9. Vrieze, A.; Holleman, F.; Serlie, M.J.; Ackermans, M.T.; Dallinga-Thie, G.M.; Groen, A.K.; van Nood, E.; Bartelsman, J.F.W.; Oozeer, R.; Zoetendal, E., et al. Metabolic effects of transplanting gut microbiota from lean donors to subjects with metabolic syndrome. *Diabetologia* **2010**, *53*, S44-S44.
10. Xi, D.; Michail, S. Fecal microbiota transplantation in children does not significantly alter body mass index. *Journal of Pediatric Gastroenterology and Nutrition* **2017**, *65 (Supplement 2)*, S73-S74, doi:http://dx.doi.org/10.1097/MPG.0000000000001805.
11. Li, S.S.; Zhu, A.; Benes, V.; Costea, P.I.; Hercog, R.; Hildebrand, F.; Huerta-Cepas, J.; Nieuwdorp, M.; Salojarvi, J.; Voigt, A.Y., et al. Durable coexistence of donor and recipient strains after fecal microbiota transplantation. *Science* **2016**, *352*, 586-589, doi:10.1126/science.aad8852.
